# Edge Zone Effect in Measurements of Asphalt Mixture Thermal Properties Using Transient Method

**DOI:** 10.3390/ma19050894

**Published:** 2026-02-27

**Authors:** Jarosław Górszczyk, Konrad Malicki

**Affiliations:** Faculty of Civil Engineering, Cracow University of Technology, 31-155 Kraków, Poland; kmalicki@pk.edu.pl

**Keywords:** asphalt mixture, thermal conductivity, specific heat capacity, thermal diffusivity, transient method, Modified Transient Plane Source (MTPS), edge zone effect, thermal properties, heat transfer

## Abstract

Thermal conductivity and specific heat capacity are key parameters controlling heat transfer and temperature distribution in road pavement structures. Although transient methods are increasingly used in laboratory testing, the thermal properties of asphalt mixtures have not been sufficiently studied using these methods, and no dedicated standards exist for road materials. This creates uncertainty in test procedures, specimen geometry, surface preparation, measurement location, and data interpretation, which may lead to significant errors, especially for massive and heterogeneous mixtures. The objective of this study is to systematically quantify the edge zone effect and assess its influence on the determined thermal parameters of a selected heterogeneous asphalt mixture. The study focuses on the quantitative determination of the edge zone effect, practical identification of its width in slab-shaped specimen, and the identification of scientific and practical methodological consequences, as well as the risks and limitations of applying the Modified Transient Plane Source (MTPS) method in the absence of standards. Laboratory measurements demonstrate a clear edge zone effect, with thermal conductivity and thermal diffusivity differing by up to 17% and 18%, respectively, near the specimen edges. These findings highlight the importance of methodological guidelines for slab-shaped asphalt mixture specimens and provide both scientific insight and practical guidance for the reliable application of transient method. They may also support the development of standardized testing procedures for asphalt mixtures.

## 1. Introduction and Problem Statement

Changes in ambient temperature influence the structural and functional characteristics of road pavements. This interaction may result in accelerated structural deterioration. Therefore, understanding the temperature distribution within a structure is crucial for designing durable and load-resistant road pavements.

The fundamental parameters used in thermal analysis of road pavement structures are the material’s thermal conductivity and specific heat capacity [1]. Various research methods are currently used to determine the thermal parameters of building materials [2], which are generally divided into two main categories: steady-state methods and transient methods [3]. The steady-state approach is standardized for insulating materials [4]. However, it requires relatively large test specimens, which creates a significant limitation in testing dense road and construction materials. In contrast, transient methods enable the testing of specimens with various shapes and dimensions. This represents one of the key differences between steady-state and transient measurement techniques [5]. A more detailed discussion of the differences between these methods can be found in the literature [2]. It should be noted, however, that no dedicated standard has yet been established for determining the thermal conductivity of road pavement materials [3]. In practice, standards developed for other materials, such as plastics [6] or rocks and soils [7], may be applied. However, it must be taken into account that due to the presence of bitumen binder, asphalt mixtures exhibit a different thermo-rheological performance than rocks or soils. Therefore, the reliable determination of thermal parameters of tested materials is essential [8]. For this reason, further research and analysis of the thermal properties of asphalt mixtures are necessary [3]. This issue has been studied since the 20th century. For example, Fwa et al. presented the laboratory determination of asphalt mixture thermal properties using the transient heat conduction method [9]. The transient method was indicated to be an attractive alternative to the steady-state method in asphalt mixture testing. Chadbourn et al. investigated the thermal behavior of asphalt mixture during compaction [10], demonstrating that the thermal conductivity of asphalt mixtures decreases as temperature increases.

In recent years, the thermal conductivity of road pavement materials and structures has been extensively investigated [11]. Mrawira and Luca examined the influence of aggregate type, gradation, and compaction level on the thermal properties of hot asphalt mixtures [12], concluding that aggregate type has the strongest effect on thermal conductivity. Luo et al. analyzed the thermal conductivity of constituent materials used in electrically heated bridge decks [13], confirming that both material composition and selected additives significantly affect the thermal performance of pavement concrete. Similar findings were reported by Abbas and Alhamdo for asphalt mixtures containing various additives [14]. Chen et al. evaluated the thermal conductivity of asphalt concrete with a heterogeneous microstructure [15]. The analysis was performed numerically using the finite element method (FEM). The authors showed that specimens that are too small lead to increased variability in the results and therefore recommended minimum specimen dimensions of 60 mm for the wearing course (aggregate grain sizes up to 12.5 mm) and 90 mm for the binding course (aggregate grain sizes up to 25 mm). This is related to the need to maintain an appropriate ratio between the specimen dimensions and the maximum aggregate grain size in the asphalt mixture. Byzyka et al. conducted a laboratory study on the thermal properties of both virgin and aged asphalt mixtures [16], observing a significant influence of temperature on thermal conductivity. Kim and Lee evaluated the heat transfer properties of asphalt mixtures by testing dry specimens as well as specimens with different levels of water saturation [17]. It was found that moisture present in the pores increases the thermal conductivity of the material. Moreover, it was reported that, for all specimen types, a reduction in layer thickness results in higher thermal conductivity. Pan et al. examined the effect of freezing–thawing and aging on the thermal characteristics and mechanical properties of conductive asphalt concrete [18], using the Transient Plane Source (TPS) method. They demonstrated that repeated freezing and thawing leads to a decrease in both the thermal conductivity and the specific heat capacity of asphalt mixtures.

Thermal properties of asphalt mixtures strongly influence both high- and low-temperature performance and, consequently, road pavement durability [19,20]. This dependence is multifactorial and results from interactions between thermal and rheological–structural phenomena. Mu et al. [21] showed that higher specimen temperatures during long-term creep tests reduce stiffness and exponentially increase rutting depth, while Al-Hamdou et al. [22] demonstrated that nanomodification improves resistance to permanent deformation and extends pavement service life at elevated temperatures. Bitumen performance plays a key role: Wang et al. [23] reported that small amounts of graphene enhance thermal stability, and Ren and Hao [24] showed that graphene reduces thermal stresses during cooling, improving low-temperature cracking resistance. Low-temperature behavior is also influenced by cooling rate and binder aging, as reported by Pszczoła and Szydłowski [25] and Li et al. [26], affecting strength reserve and cracking susceptibility.

At the same time, research methods are evolving, particularly in the field of transient techniques. For example, Malinarić et al. proposed a numerically enhanced transient method for evaluating the thermal properties of anisotropic materials [27]. Zheng et al. conducted an analysis and improvement in the hot disk TPS method for materials with low thermal conductivity [28]. The results of these studies indicate that improving measurement methods and instrumentation can lead to more precise results, particularly for anisotropic materials.

Asphalt mixture specimens can be produced in various shapes and dimensions, most commonly cylindrical or slab-shaped. In road materials laboratories, slab-shaped specimens obtained via the rolling method, typically with surface dimensions of approximately 300 × 300 mm^2^, are often used. It should be noted that, due to the absence of a standardized procedure for determining the thermal parameters of asphalt mixtures, no standard specimen shapes or minimum dimensions have been defined, unlike in Transient Line Source (TLS) testing of soils and rocks [7]. This raises concerns regarding the variability of thermal parameter values at different locations within the specimen. This issue is related to the so-called edge zone effect and the heterogeneity of the material.

The objective of this study is to evaluate the influence of the edge zone effect on the determined thermal properties of a selected asphalt mixture, including thermal conductivity, specific heat capacity, and thermal diffusivity. Additionally, the influence of the specimen surface condition on thermal conductivity was assessed. However, the novelty of the present study does not lie only in identifying the existence of the edge zone effect, but also in:

- The quantitative determination of the edge zone effect for the selected real, heterogeneous asphalt mixture;
- The practical determination of the width of the edge zone for the asphalt mixture prepared as a slab-shaped specimen used in road materials laboratories;
- Identifying the scientific and practical methodological consequences, as well as the risks and limitations associated with the application of the Modified Transient Plane Source (MTPS) method to the slab-shaped specimen in the absence of dedicated standards.

The tests were conducted using the MTPS method, with the probe positioned at multiple locations on the specimen surface to allow analysis of spatial variation in thermal properties. The obtained results made it possible to determine the differences between parameters calculated in the central part and the edge zone of the specimen. Moreover, the results allowed for an assessment of the significance of the edge zone effect for the reliability of measurements performed using a transient method for the selected asphalt mixture.

## 2. Research Method and Theoretical Model

Thermal properties of the asphalt mixtures were tested in laboratory conditions using a device based on the MTPS method [29]. This method belongs to the group of transient methods and allows the simultaneous determination of thermal conductivity (λ), specific heat capacity (c_p_), and thermal diffusivity (a) based on the analysis of short-term heat flow generated by a thermal pulse.

In the MTPS method, the flat sensor acts both as a heat source and a temperature detector. When the sensor is placed on the specimen surface, a known heat flux $q\;(W\cdot m^{-2})$ is applied, and the temperature change at the contact point is described by the heat conduction Equation (1) for a semi-infinite, isotropic medium in cylindrical coordinates, assuming axial symmetry [30]:

(1) $$\frac{\partial T}{\partial t}=a\left(\frac{1}{r}\frac{\partial }{\partial r}\left(r\frac{\partial T}{\partial r}\right)+\frac{\partial^{2}T}{\partial z^{2}}\right),$$

where

r—the radial coordinate (distance from the center of the sensor),
z—the depth coordinate measured perpendicular to the specimen surface,
t—time,
T(r,z,t)—temperature (K),
$a=\lambda \cdot \rho^{-1}\cdot c_{p}^{-1}$—thermal diffusivity ${(m}^{2}\cdot s^{-1})$,
λ—thermal conductivity $\left(W\cdot m^{-1}\cdot K^{-1}\right)$,
ρ—density $\left(kg\cdot m^{-3}\right)$,
$c_{p}$—specific heat capacity $\left(J\cdot {kg}^{-1}\cdot K^{-1}\right)$,
$c_{v}=\rho \cdot c_{p}$—volumetric heat capacity $\left(J\cdot m^{-3}\cdot K^{-1}\right)$.

To represent pulsed heating over a limited surface area of a semi-infinite medium, appropriate initial and boundary conditions were assumed.

Initial condition:

(2) $$T\left(r,z,0\right)=T_{0}$$

where $T_{0}$ is the uniform initial temperature of the specimen.

Boundary conditions:

- At the specimen surface, in the area of contact with the MTPS sensor, a known short-time heat pulse is applied. It is described by the heat flux:

(3) $$q\left(r,0,t\right)=q_{0}\left(r\right)H(t)$$

where $q_{0}\left(r\right)$ is the spatial distribution of the heat flux resulting from the sensor geometry, H(t) is the time function of the heat pulse with a finite duration. Outside the sensor contact area, the heat flux is assumed to be zero.

- The specimen is assumed to behave as a semi-infinite medium in the direction normal to its surface:

(4) $$T\left(r,z\to \infty ,t\right)=T_{0}$$

which means that, during the measurement time, the thermal wave does not reach the bottom boundary of the specimen.

- Radial condition reflects the assumption that the lateral boundaries of the specimen are sufficiently far away so and do not influence the local temperature response in the measurement area:

(5) $$T\left(r\to \infty ,z,t\right)=T_{0}$$

Under these assumptions, an analytical solution of Equation (1) can be obtained. The resulting temperature increase at the center of the MTPS sensor, $∆T=T(0,0,t)-T_{0}$, can be expressed in a general functional form as a function of time, material thermal properties, and the applied heat flux [30]:

(6) $$∆T(0,0,t)=f(t,\lambda ,c_{v},q)$$

The parameters λ, $c_{v}$ are determined in the ISOMET 2114 (MTPS) device using an inverse (optimization) procedure. This procedure fits the theoretical model to the measured temperature response. The difference between the calculated and experimental signals is minimized, which allows the thermal properties of the tested materials to be determined in a repeatable manner. The thermal diffusivity is subsequently calculated from the obtained values of thermal conductivity and volumetric heat capacity as their ratio.

The adopted model is based on the assumption of a locally semi-infinite medium. This assumption is satisfied only when the characteristic thermal penetration depth is much smaller than the specimen thickness and the distance from the measurement point to the lateral edges. Violation of this assumption in the near-edge region leads to the occurrence of the so-called edge-zone effect, which is the subject of the present analysis.

## 3. Tested Material and Measurement Procedure

### 3.1. Tested Material

Laboratory tests were carried out on a selected asphalt mixture typically used in road pavements for the binding course. This mixture was selected due to the medium aggregate size typically applied in this type of asphalt mixture. A designation and basic parameters of the asphalt mixture are presented in Table 1.

**Figure 1:**
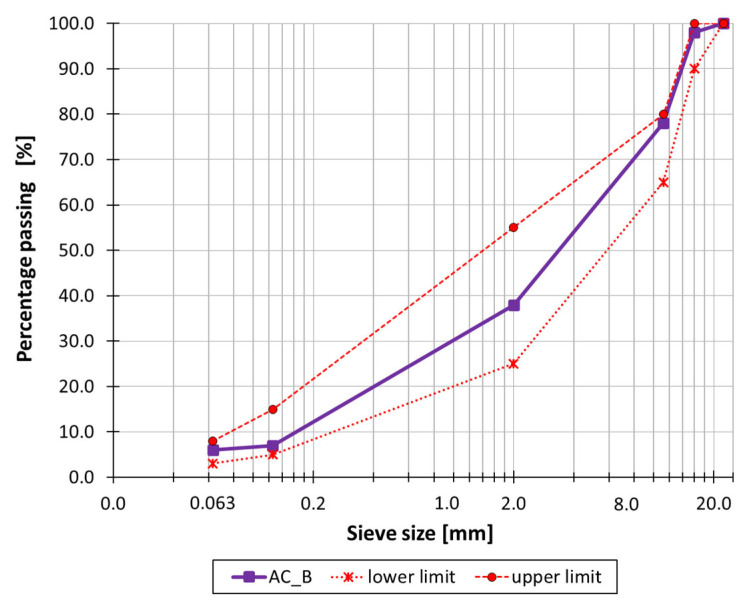
Gradation curve along with upper and lower limit curves.

The binder used in the asphalt mixture was 50/70 penetration grade bitumen, with a bitumen content of 4.81% [m/m]. Limestone filler and limestone aggregate were used in the mineral mixture. The grading curve of the mineral mixture used in the asphalt mixture, along with the upper and lower limit curves, are shown in Figure 1.

The asphalt mixture was prepared in a laboratory mixer (Bau-stoff-Prufsysteme Wennigsen GmbH, Wennigsen, Germany) while maintaining the required process temperatures [31]. Cylindrical Marshall specimens were first produced using the Marshall impact compaction method. Specimens were compacted with 2 × 50 blows. The compaction temperature was equal to 145 ± 5 °C. These specimens were used to determine the physical parameters of the asphalt mixture. The obtained results were compared to the national technical requirements for asphalt pavements [31], and all requirements were met. The results, along with the relevant test standards, are summarized in Table 2.

**Figure 2:**
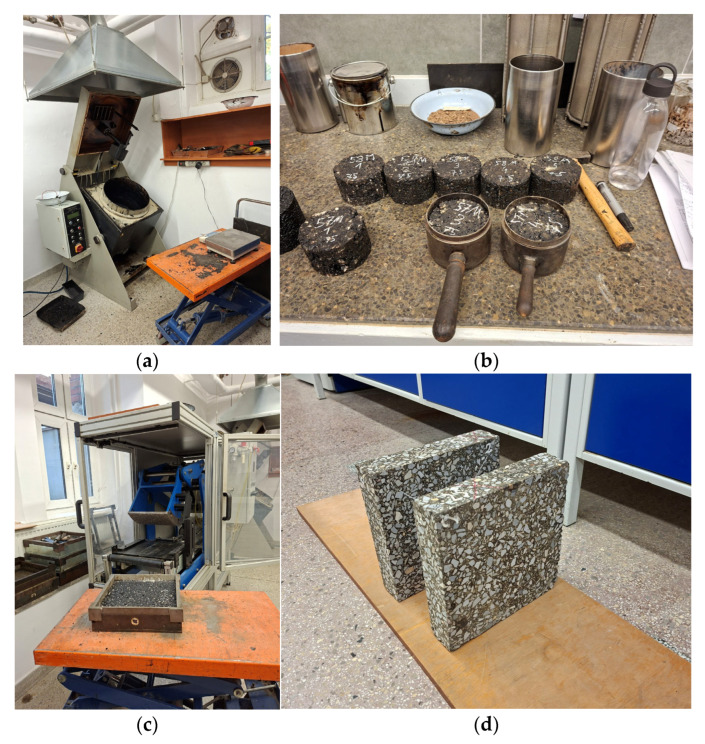
Preparation of asphalt mixture specimens: (**a**) mixing in a laboratory mixer; (**b**) Marshall specimens; (**c**) slab specimen in the mold after roller compaction; (**d**) slab specimens after trimming.

In the next step, two slab specimens with dimensions of 30.5 × 30.5 × 10.0 cm^3^ were prepared [35]. The slab specimens were compacted using a roller compactor (Cooper Research Technology, Ripley, UK). After demolding, the slabs underwent preliminary tests and were then cut to final dimensions of 28.5 × 28.5 × 6.0 cm^3^, ensuring uniform and flat side surfaces. Thermal parameter measurements were conducted on the first slab, while the second slab was used for preliminary tests and equipment verification. The specimen preparation process is illustrated in Figure 2.

### 3.2. Measurement Procedure and Experimental Program

**Figure 3:**
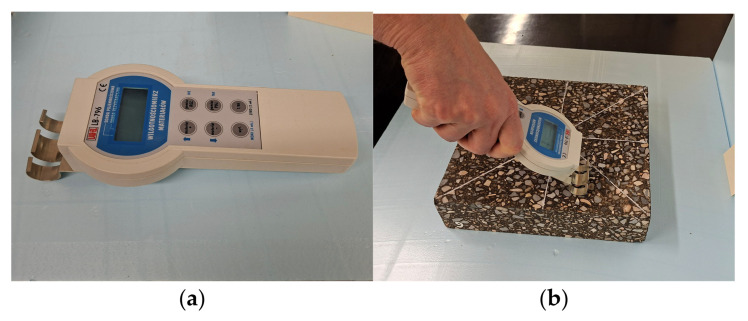
Moisture testing of the specimen: (**a**) moisture meter type LB-796; (**b**) moisture measurement using capacitive method.

Before testing, the slab-shaped specimens were conditioned at laboratory room temperature for 72 h. This procedure ensured the stabilization of both temperature and moisture content within the specimens. During the thermal parameter measurements, air temperature, air humidity, and specimens moisture were monitored. The air temperature was maintained at +21 ± 1 °C, while the air humidity was 37% ± 5%. The moisture content of the specimens was 1.4% ± 0.2%. The specimens moisture was measured using a capacitive method, which determines the dielectric constant of the tested material assuming a known material density. A material moisture meter type LB-796 (LAB-EL Electronics Laboratory Sp. z o.o., Reguly, Poland) was used for this purpose. The moisture meter and the moisture measurement are shown in Figure 3.

The Isomet 2114 device (Applied Precision Ltd., Bratislava, Slovakia), operating on the transient method, was used to determine the thermal parameters of the material [29]. The device is equipped with a modified surface probe IPS 1105 (Applied Precision Ltd., Bratislava, Slovakia) designed for testing hard materials using the MTPS method. This method enables measurements on a single surface of the specimen, eliminating the need to place the probe between two surfaces, as required in the TPS method. The MTPS probe functions by inducing and recording temperature changes over time on the tested surface. The device operates within a temperature range from −15 to +50 °C, and it allows the determination of thermal conductivity in the range of 0.04–6.0 W·m^−1^·K^−1^. The accuracy of thermal conductivity measurements is 10% of the reading, while for volumetric heat capacity measurements it is 15% of the reading +1.10^3^ J·m^−3^·K^−1^. The reproducibility of the measurements is 3% of the reading +0.001 W·m^−1^·K^−1^ for thermal conductivity and 3% of the reading +1.10^3^ J·m^−3^·K^−1^ for volumetric heat capacity [29].

**Figure 4:**
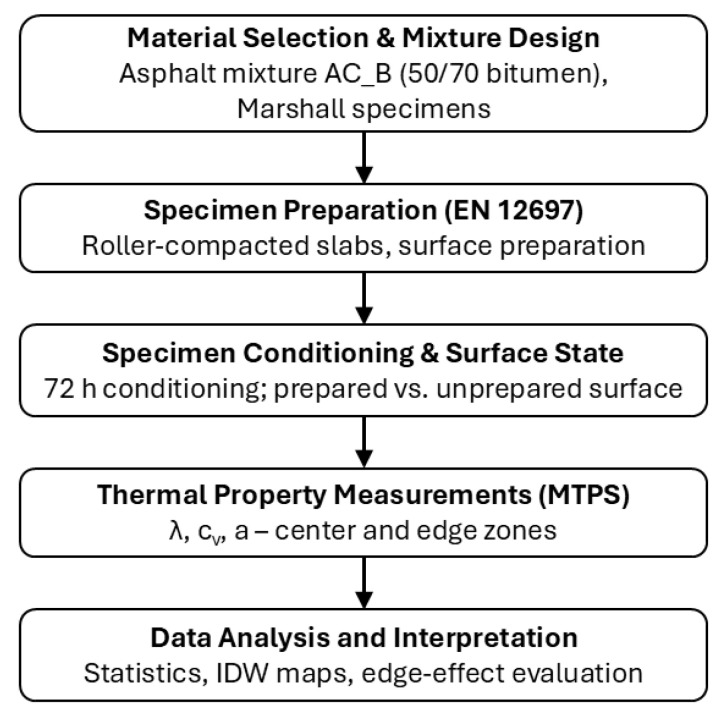
Flowchart of the experimental program used to evaluate the spatial variability of thermal properties of selected asphalt mixture slab-shaped specimen.

The experimental program involved testing the thermal parameters of the asphalt mixture while considering different probe locations on the specimen surface, both in the central area and near the edges, to assess the edge zone effect. The experimental research program, presented in a flowchart format, is shown in Figure 4.

**Figure 5:**
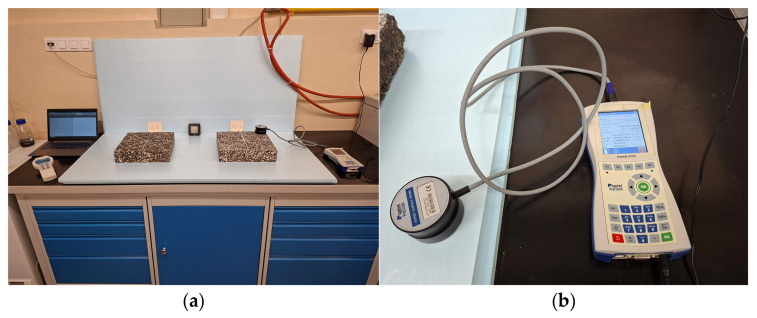

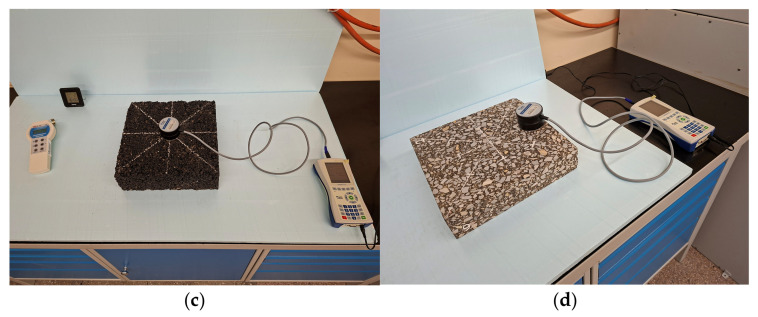
Thermal parameter testing of the asphalt mixture: (**a**) laboratory testing setup; (**b**) Isomet 2114 device; (**c**) measurement on the top surface of the asphalt mixture specimen before trimming at measurement point No. 0*; (**d**) main tests—measurement on the top, trimmed surface of the specimen at the designated measurement point.

**Figure 6:**
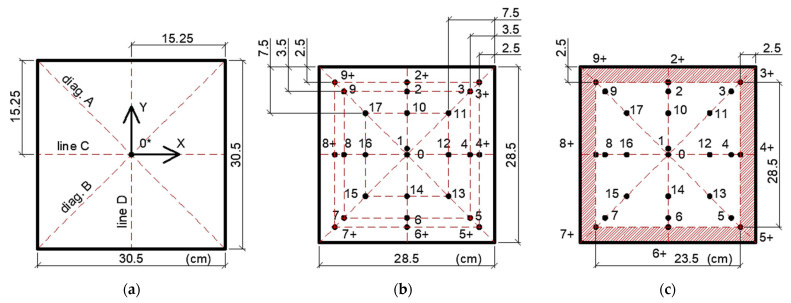
Layout and designation of measurement points on the top surface of the specimen: (**a**) measurement on specimen before cutting; (**b**) measurement points on the specimen after cutting. Measurement lines are marked in red; (**c**) area where the probe cannot be positioned without extending its edge beyond the specimen edge (red hatched area). The white area shows the region used to prepare 2D maps of the thermal parameters. Dimensions related to the distribution of the points (measurement lines) are given in centimeters.

Prior to the main tests, the influence of specimen surface preparation on the measurement results was evaluated. Proper operation of the Isomet device requires full thermal contact between the measuring probe and the specimen surface. However, the surface of a typical laboratory-prepared asphalt mixture specimen (similar to the surface of the asphalt mixtures used in road pavements) exhibits a macrotexture. As a result, the probe rests on individual aggregate particles rather than making full-surface contact, which affects the determined parameter values. To assess this effect, thermal parameter results obtained at a fixed point on the slab specimen before surface preparation (point 0*) (Figure 5c and Figure 6a) were compared with results obtained after the specimen was trimmed and smoothed (point 0). The test setup and the procedure for measuring thermal parameters on the top surface of the specimen are shown in Figure 5.

The primary investigations concerned the spatial variability of the thermal parameter values on the trimmed and prepared surface of the slab-shaped specimen (Figure 5d). On this surface, 26 measurement points were designated. Five measurements were taken at each point. In total, 130 measurements were performed on the top surface of the specimen. The layout and designation of the measurement points are shown in Figure 6. Points marked with “+” indicate the locations farthest from the center of the specimen. In Figure 6c, the red color indicates the region near the specimen edge where the probe cannot be positioned without extending its edge beyond the specimen edge.

## 4. Results and Discussion

### 4.1. Influence of Specimen Surface Condition

In the first stage, the study examined whether the condition of the specimen surface affects the determined thermal parameters. For the uncut specimen with an unprepared surface at measurement point 0*, the average thermal conductivity was 1.727 ± 0.003 W·m^−1^·K^−1^. This value was significantly lower than the average thermal conductivity obtained at the same point (point 0) on the cut specimen (λ = 2.001 ± 0.004 W·m^−1^·K^−1^). The rough surface texture reduced the thermal contact between the sensor and the material, resulting in an underestimated measurement.

To confirm the significance of the differences between the cut and uncut surfaces, an analysis of variance (ANOVA) was performed. First, the assumptions of the analysis were verified. The normality of the distributions was confirmed using the Shapiro–Wilk test at the significance level α = 0.05, and the homogeneity of variances was confirmed using Levene’s test. Statistically significant differences were then evaluated using the Scheffé post hoc test. For α = 0.05, the results showed statistically significant differences. Therefore, the surface condition has a notable influence on the correct determination of thermal parameters of asphalt mixture.

For this reason, all thermal parameters in the main part of the study were determined on cut and properly prepared specimen surfaces.

### 4.2. Main Test Results

The obtained results are summarized in Table 3, and the arrangement of measurement points is shown in Figure 6. Point 0* refers to the measurement conducted at the exact location of point 0, but on a specimen with an unprepared surface.

The mean thermal parameters obtained in the laboratory tests were used to generate 2D maps of their distribution on the top surface of the specimen (Figure 6c). These maps were developed using Inverse Distance Weighting (IDW) interpolation. The standard power parameter value of *p* = 2 was used in the analysis, as it is most commonly applied in engineering studies and offers a compromise between smoothing the field and maintaining local extremes.

### 4.3. Discussion of Thermal Conductivity Results

**Figure 7:**
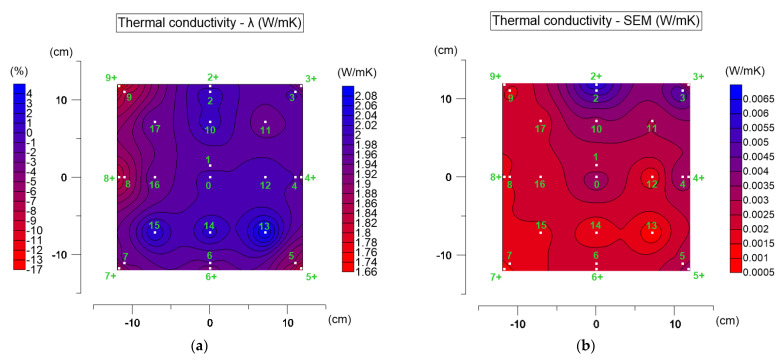
Thermal conductivity test results: (**a**) distribution of mean values on the top surface of the AC_B asphalt mixture specimen (right scale) and relative differences compared to the central point (point 0, left scale); (**b**) distribution of the standard errors of the mean (SEM).

Figure 7 presents the distribution of average thermal conductivity values on the specimen surface (Figure 7a) and the corresponding distribution of standard errors of the mean (Figure 7b). In Figure 7a, an additional percentage scale (left side) shows the variation in thermal conductivity in each point relative to the central point (measurement point 0).

For the tested asphalt mixture, thermal conductivity ranged from 1.661 to 2.100 W·m^−1^·K^−1^, which is consistent with values reported in the literature [12]. A clear spatial variation in the parameter is observed. The most uniform values occur in the central part of the specimen, where thermal conductivity is approximately 2.0 W·m^−1^·K^−1^. In some points of the second measurement line (points 13, 14, 15), conductivity locally increased to about 2.1 W·m^−1^·K^−1^, which did not occur in other points of the same line (e.g., 11, 16, 17). This suggests the presence of local material heterogeneity.

A distinct edge zone effect is also visible, especially within about 4 cm from the specimen boundary. Many edge points (e.g., 3+, 5+, 7+, 8+, 9+) showed reduced thermal conductivity values around 1.7 W·m^−1^·K^−1^. This behavior is expected: due to lateral heat transfer by convection along the side surfaces of the specimen, the thermal parameters in the edge zone differ from those in the central region. The differences reach up to about 17%.

The highest standard errors of the mean (Figure 7b) were observed near points 2 and 3. Although they reached values up to 0.008 W·m^−1^·K^−1^, they were still within the repeatability range declared by the device manufacturer.

The thermal conductivity obtained in the central part (point 0) was taken as representative for the material. To verify whether the edge values differ significantly from the central value, ANOVA was conducted. The assumptions of normal distribution (Shapiro–Wilk test) and homogeneity of variances (Levene’s test) were confirmed. The Scheffé post hoc test was then applied. At α = 0.05, most edge points differed significantly from the central point (except point 4).

As shown in Figure 7a, the values at points 14 and 15 are approximately 2.05 W/mK, resulting in a difference of about 2%. At point 13, the thermal conductivity is approximately 2.1 W/mK, corresponding to a difference of about 5%. Although this difference is statistically significant, its magnitude is relatively small (not exceeding 5%). Such variations may result from material heterogeneity. More pronounced differences are observed at the corners and near the specimen edges (e.g., points 3+, 5+, 7+, 8+, and 9+). These differences reach up to 17%. Such large deviations cannot be explained only by specimen heterogeneity and confirm the influence of the edge zone effect. The effects of material heterogeneity and the edge zone effect may partially overlap in certain locations.

The analysis clearly shows that the edge zone effect significantly influences thermal conductivity measurements, and it must be considered when interpreting results obtained using transient methods.

### 4.4. Discussion of Volumetric Heat Capacity Results

**Figure 8:**
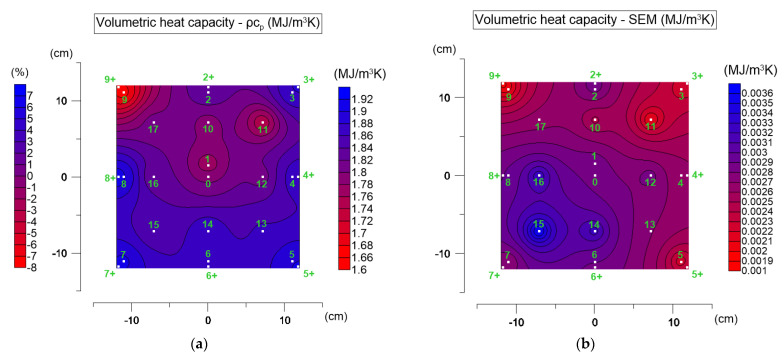
Volumetric heat capacity test results: (**a**) distribution of mean values on the top surface of the AC_B asphalt mixture specimen (right scale) and relative differences compared to the central point (point 0, left scale); (**b**) distribution of the standard errors of the mean (SEM).

Figure 8 shows the average distribution of volumetric heat capacity on the specimen surface (Figure 8a) and the corresponding standard errors of the mean (Figure 8b). In Figure 8a, two scales are presented: the right scale shows absolute values of volumetric heat capacity, while the additional scale shows percentage differences relative to the central point (point 0).

For the tested asphalt mixture, volumetric heat capacity ranged from 1.657 to 1.934 MJ·m^−3^·K^−1^. When converted to specific heat capacity, the values range from 0.691 kJ·kg^−1^·K^−1^ to 0.807 kJ·kg^−1^·K^−1^. These results are consistent with the values reported in the literature [16]. The spatial variability of this parameter is clearly smaller than that of thermal conductivity. The most stable values appear near points 13, 14, and 15. The largest deviation occurs at the corner (point 9+), with the lowest value—1.657 MJ·m^−3^·K^−1^.

The differences between the central and edge areas reach up to about 8%. The highest standard errors (Figure 8b) occur near points 14, 15, and 16, with values up to 0.006 MJ·m^−3^·K^−1^, which still remain within the device’s declared repeatability. The volumetric heat capacity at point 0 was taken as representative. ANOVA was performed to verify whether differences between the central point and the edge zone were statistically significant. At α = 0.05, all assumptions were satisfied, and the Scheffé post hoc test confirmed significant differences between the central value and the edge region.

With regard to volumetric heat capacity, it should be noted that in the MTPS method this parameter is not determined independently. It is obtained through an inverse fitting procedure, in which thermal conductivity and volumetric heat capacity are estimated simultaneously. When the semi-infinite medium assumption is not fully satisfied, additional lateral heat losses may influence the temperature–time response. In such cases, the inverse solution may adjust both parameters in a coupled manner. This coupling may partially explain the observed increase in volumetric heat capacity near specimen edges. At the same time, local density variations and material heterogeneity may also influence volumetric heat capacity, since it depends directly on material density.

Thus, the analysis shows that the edge zone effect also influences volumetric heat capacity measurements, although to a smaller extent than thermal conductivity.

### 4.5. Discussion of Thermal Diffusivity Results

**Figure 9:**
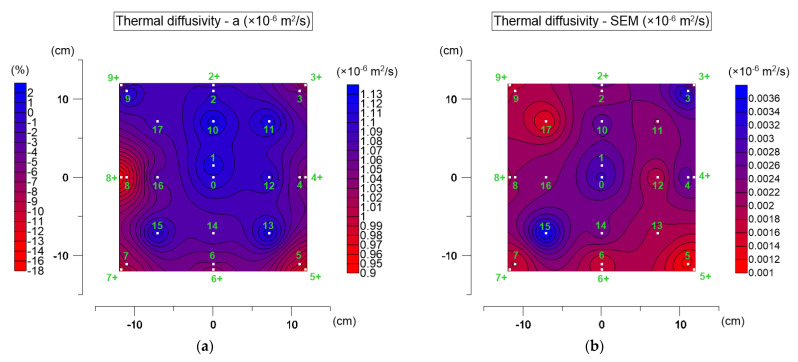
Thermal diffusivity test results: (**a**) distribution of mean values on the top surface of the AC_B asphalt mixture specimen (right scale) and relative differences compared to the central point (point 0, left scale); (**b**) distribution of the standard errors of the mean (SEM).

Figure 9 shows the distribution of average thermal diffusivity values on the specimen surface (Figure 9a) and the corresponding standard errors of the mean (Figure 9b). In Figure 9a, the right scale presents absolute diffusivity values, while the left scale shows differences relative to the central point (point 0).

For the tested asphalt mixture, thermal diffusivity ranged from 0.910 to 1.161·10^−6^ m^2^·s^−1^, with a value of about 1.110·10^−6^ m^2^·s^−1^ in the central part. This range agrees with values reported in the literature [36]. A clear spatial variation is visible, reflecting local heterogeneity of the material. The most characteristic feature is the reduction in diffusivity in the edge zone—particularly at points 5+, 7+, and 8+, where values drop to approximately 0.9·10^−6^ m^2^·s^−1^. The differences between the central and edge zones reach up to 18%. Such non-uniformity may result from edge losses due to convection. The highest standard errors (Figure 9b) were recorded near point 15, reaching 0.004·10^−6^ m^2^·s^−1^, which is within the declared repeatability. The diffusivity value at point 0 was taken as representative. ANOVA was used to verify differences between central and edge regions. At α = 0.05, normality (Shapiro–Wilk test) and homogeneity of variance (Levene’s test) were confirmed. The Scheffé post hoc test indicated statistically significant differences, confirming the influence of the edge zone effect.

### 4.6. Discussion Summary and Implications

In the first stage, the obtained research results were compared with literature data. A comparison of the obtained results, with selected results reported by other researchers, is presented in Table 4.

It should be noted that the literature studies involved different types of asphalt mixtures, specimen geometries, and were conducted under various conditions. However, the authors did not find any publications addressing the variability of thermal parameters of asphalt mixtures tested on slab specimens. Therefore, the comparison can only be general and not refer to exact values. Taking these aspects into account, it can be stated that the results obtained in this study are consistent with those reported in the literature.

The obtained results were analyzed in relation to typical uncertainty ranges in thermal modelling of pavement structures and to the sensitivity of pavement temperature predictions. The discussion also considered whether the observed differences are important from an engineering point of view. In addition, the selection of the central point as a reliable reference location was evaluated.

Abbas and Alhamdo reported that differences in the thermal conductivity λ of 2–4% can be treated as comparable [14]. Chu et al. showed that the average errors of calculation models used to estimate the thermal conductivity of asphalt mixtures usually range from 4.25% to 11.45%, depending on the mixture type [37]. Compared with these values, the laboratory differences in thermal parameters observed in this study, equal to 17–18%, indicate clear differences in the functional performance of the material.

Malinaric et al. demonstrated that the modified dynamic plane source method provides measurement errors below 0.8% for anisotropic specimens with a thickness of 9 mm and thermal conductivity below 2 W·m^−1^·K^−1^ over the full thickness [27]. In the present study, SEM values did not exceed 0.8%, which confirms good repeatability of the results, especially in the central part of the specimens.

**Figure 10:**
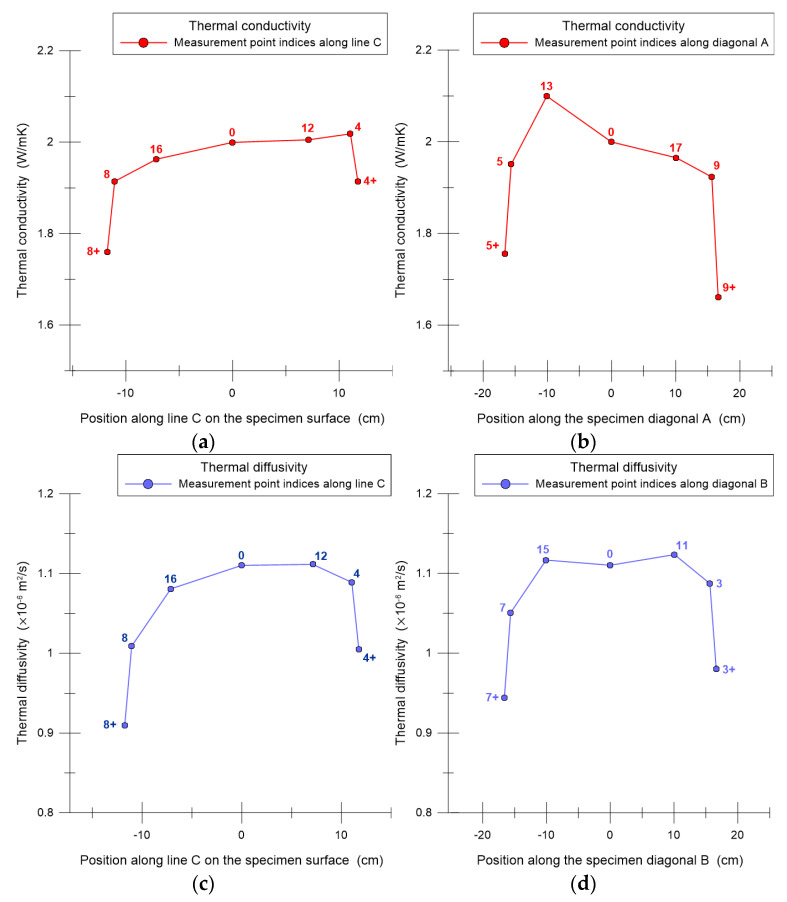
Thermal parameter measurement results along selected lines (Figure 6a): (**a**) thermal conductivity determined along line C; (**b**) thermal conductivity determined along diagonal A; (**c**) thermal diffusivity determined along line C; (**d**) thermal diffusivity determined along diagonal B.

A considerably lower variability of results was observed in the central part of the specimen than in the edge zones, which supports the selection of point 0 as a reliable reference location. The results indicate that measurements taken close to the specimen edges are influenced by edge zone effects. These effects are mainly related to heat losses caused by natural convection, which lead to a systematic underestimation of thermal conductivity and thermal diffusivity compared with measurements performed in the central area. This behavior is clearly illustrated in Figure 10.

These findings have direct implications for pavement temperature modelling. Omidvar et al. showed that pavement temperature predictions are very sensitive to the assumed thermal conductivity of pavement materials, as changes in the range from 0.3 to 3.1 W·m^−1^·K^−1^ may reduce the pavement surface temperature by almost 10 °C [38]. In this context, the 17–18% differences in thermal conductivity observed in this study should be considered important from an engineering point of view, as they may strongly influence the results of thermal analyses of full pavement structures. Furthermore, according to the EN IEC 60751 standard [39] and typical Pt100 sensor specifications [40], the temperature measurement error for class A sensors is within ±(0.15 + 0.002·t) °C. This suggests that the potential impact of variability in thermal properties on modeled temperature fields may exceed the intrinsic accuracy of commonly used pavement temperature sensors. This confirms the need to determine representative thermal conductivity values for asphalt mixtures and to develop standard laboratory testing procedures based on transient methods. The key scientific and practical aspects, as well as the limitations and risks associated with laboratory tests conducted using the transient method (MTPS), are summarized in Table 5.

Accurate thermal data are essential for thermal analyses of entire pavement structures [41]. They are also important for the proper selection of road materials, especially with regard to heat accumulation and its negative effects on the environment and human health, which are related to the urban heat island (UHI) phenomenon [42]. Therefore, further research is needed to address the identified limitations and risks, including studies on different asphalt mixtures and a wider range of testing conditions, such as temperature and moisture. This will allow for a better assessment of the suitability of the MTPS method for road material testing.

## 5. Conclusions

The following conclusions are drawn from the conducted research:

- The MTPS method makes it possible to effectively and repeatably determine the thermal parameters of slab-shaped asphalt mixture specimens, allowing the evaluation of their variability at different points on the specimen surface.
- The surface condition of the specimen has a significant influence on the measurement results. The surface used for testing should be as flat as possible and carefully cleaned to reduce measurement errors.
- For the tested asphalt mixture, a clear spatial variability of thermal parameters is observed on the specimen surface. For thermal conductivity, values ranged from 1.66 to 2.10 W·m^−1^·K^−1^. The variation in the central part of the specimen may result from material heterogeneity, while a distinct edge zone effect appears near the specimen boundary. This effect is related to additional heat transfer by convection along the side surfaces of the specimen, which leads to a systematic underestimation of locally determined thermal conductivity and thermal diffusivity.
- In the edge zone, clear deviations in thermal parameters were recorded: thermal conductivity values were up to about 17% lower, and thermal diffusivity was up to about 18% lower compared with the central part of the specimen. For volumetric heat capacity, an opposite trend was observed—in most edge-zone locations, values were higher than in the center.
- For the tested specimen, the edge zone can be defined as the region extending up to approximately 4 cm inward from the boundary of the asphalt mixture slab.
- The ANOVA analysis confirmed a statistically significant influence of the edge zone effect on the determined thermal parameters (α = 0.05).
- The results indicate that, due to the strong influence of the edge zone effect, the thermal parameters of asphalt mixtures should not be determined from measurements taken within the edge zone of the slab-shaped specimen.
- The studies made it possible to identify a number of scientific and practical risks and limitations related to the application of the MTPS method in the testing of asphalt mixture slab-shaped specimen. The identified limitations require further research and analysis.
- The results obtained refer to a single selected asphalt mixture and should be considered as a case study. Therefore, they cannot be generalized to other types of asphalt mixtures. These findings provide a starting point for the discussion of the investigated issue and highlight the need for further research, including other mixture types and specimen geometries.

## Figures and Tables

**Table 1:** Basic parameters of the asphalt mixture.

Asphalt Mixture Designation	Asphalt Mixture Type	Location in Road Pavement Structure	Maximum Aggregate Size [mm]
AC_B	asphalt concrete	binding course	16

**Table 2:** Asphalt mixture tests results.

Parameter	Results	Requirements [31]	Standard
Bulk density (mg∙m^−3^)	2.397	—	EN 12697-6 [32]
Density (mg∙m^−3^)	2.498	—	EN 12697-5 [33]
Voids filled with bitumen VFB (%)	73.9	60–80	EN 12697-8 [34]
Voids of mineral aggregate VMA (%)	15.5	min. 14	EN 12697-8 [34]
Void ratio VV (%)	4.0	3.0–6.0	EN 12697-8 [34]

**Table 3:** Mean thermal parameters of the tested AC_B asphalt mixture, together with the standard errors of the mean (SEM). The temperature difference during all measurements was ΔT = 9.9 °C (mean value).

Measuring Point Number	Test Temperature	Thermal Conductivity	Volumetric Heat Capacity	Thermal Diffusivity
	T_mean_ (°C)	λ (W∙m^−1^∙K^−1^)	c_v_ (MJ∙m^−3^∙K^−1^)	a (×10^−6^ m^2^∙s^−1^)
	Mean	Mean ± SEM	Mean ± SEM	Mean ± SEM
0	27.4	2.001 ± 0.004	1.801 ± 0.003	1.110 ± 0.003
1	26.6	1.966 ± 0.002	1.743 ± 0.003	1.128 ± 0.003
2	27.6	2.059 ± 0.004	1.835 ± 0.003	1.122 ± 0.002
3	26.9	2.041 ± 0.007	1.877 ± 0.002	1.087 ± 0.004
4	27.2	2.018 ± 0.004	1.853 ± 0.003	1.089 ± 0.003
5	27.2	1.951 ± 0.002	1.894 ± 0.001	1.030 ± 0.001
6	27.1	2.018 ± 0.002	1.872 ± 0.003	1.078 ± 0.001
7	27.1	2.006 ± 0.002	1.909 ± 0.002	1.051 ± 0.001
8	26.3	1.914 ± 0.003	1.897 ± 0.003	1.009 ± 0.002
9	26.6	1.923 ± 0.002	1.657 ± 0.002	1.161 ± 0.002
10	26.6	2.023 ± 0.003	1.785 ± 0.002	1.133 ± 0.003
11	26.7	1.940 ± 0.003	1.726 ± 0.001	1.124 ± 0.002
12	26.7	2.005 ± 0.001	1.804 ± 0.003	1.111 ± 0.002
13	26.7	2.100 ± 0.001	1.863 ± 0.003	1.127 ± 0.002
14	26.8	2.046 ± 0.001	1.887 ± 0.004	1.084 ± 0.003
15	26.7	2.055 ± 0.002	1.841 ± 0.005	1.117 ± 0.004
16	26.7	1.963 ± 0.002	1.816 ± 0.004	1.081 ± 0.002
17	26.8	1.965 ± 0.002	1.809 ± 0.003	1.086 ± 0.001
2+	27.3	1.956 ± 0.008	1.842 ± 0.003	1.062 ± 0.003
3+	27.1	1.856 ± 0.001	1.893 ± 0.002	0.980 ± 0.001
4+	27.1	1.914 ± 0.002	1.904 ± 0.003	1.005 ± 0.002
5+	27.3	1.755 ± 0.004	1.913 ± 0.003	0.917 ± 0.002
6+	27.2	1.858 ± 0.002	1.859 ± 0.002	1.000 ± 0.002
7+	27.2	1.796 ± 0.001	1.903 ± 0.003	0.944 ± 0.002
8+	27.1	1.760 ± 0.001	1.934 ± 0.003	0.910 ± 0.001
9+	27.2	1.661± 0.002	1.670 ± 0.001	0.995 ± 0.001
0*	27.1	1.727 ± 0.003	1.530 ± 0.003	1.129 ± 0.001

**Table 4:** Comparison of the obtained results with selected results reported by other researchers.

Parameter	Range of Results Obtained in this Study for Different Measurement Points	Selected Approximate Values Reported by Other Researchers and Remarks	Type of Specimens Used by Other Researchers	Ref.
Thermal conductivity [W·m^−1^·K^−1^]	1.661–2.100	1.7–2.1 for different asphalt mixtures and testing conditions	Cylindrical specimens compacted using a gyratory compactor	[12]
		1.1–2.0 for different asphalt mixtures and testing conditions	Cylindrical Marshall specimens	[16]
Specific heat capacity [kJ·kg^−1^·K^−1^]	0.691–0.807	0.7–1.4 for different asphalt mixtures and testing conditions	Cylindrical Marshall specimens	[16]
Thermal diffusivity [×10^−6^ m^2^·s^−1^]	0.910–1.161	1.24–1.27 for asphalt mixtures with conductive fillers	Cylindrical Marshall specimens	[36]
		0.4–1.1 for different asphalt mixtures and testing conditions	Cylindrical Marshall specimens	[16]

**Table 5:** Key scientific and practical aspects, limitations, and risks associated with the obtained results.

Domain	Aspect	Main Findings	Limitations and Risks
Scientific	Spatial variability	Significant local variability of thermal parameters within a single specimen was identified	Results may vary depending on the measurement point location
Scientific	Edge zone effect	Thermal conductivity at specimen edges was up to approximately 17% lower than in the central region (thermal diffusivity up to 18%)	Risk of systematic underestimation/overestimation if measurement location is not controlled
Scientific	Measurement method	The MTPS method reveals material heterogeneity	Sensitivity to local variations in material composition
Practical	Measurement procedures for various surface conditions	Standardization of surface preparation is required	Limited repeatability without control of surface condition
Practical	Practical applicability to large slab specimen testing	The method is suitable for scientific research	Standardization of measurement procedures is required for routine quality control of slab-shaped specimens

## Data Availability

The original contributions presented in the study are included in the article. Further inquiries can be directed to the corresponding author.
